# Investigating the Influence of Magnesium Ions on p53–DNA Binding Using Atomic Force Microscopy

**DOI:** 10.3390/ijms18071585

**Published:** 2017-07-21

**Authors:** Yang Chen, Tianyong Gao, Yanwei Wang, Guangcan Yang

**Affiliations:** School of Physics and Electronic Information, Wenzhou University, Wenzhou 325035, China; speedmatrix2006@gmail.com (Y.C.); 16451183154@wzu.edu.cn (T.G.); wangyw@wzu.edu.cn (Y.W.)

**Keywords:** p53–DNA interaction, metal ions, atomic force microscopy

## Abstract

p53 is a tumor suppressor protein that plays a significant role in apoptosis and senescence, preserving genomic stability, and preventing oncogene expression. Metal ions, such as magnesium and zinc ions, have important influences on p53–DNA interactions for stabilizing the structure of the protein and enhancing its affinity to DNA. In the present study, we systematically investigated the interaction of full length human protein p53 with DNA in metal ion solution by atomic force microscopy (AFM). The p53–DNA complexes at various p53 concentrations were scanned by AFM and their images are used to measure the dissociation constant of p53–DNA binding by a statistical method. We found that the dissociation constant of p53 binding DNA is 328.02 nmol/L in physiological buffer conditions. The influence of magnesium ions on p53–DNA binding was studied by AFM at various ion strengths through visualization. We found that magnesium ions significantly stimulate the binding of the protein to DNA in a sequence-independent manner, different from that stimulated by zinc. Furthermore, the high concentrations of magnesium ions can promote p53 aggregation and even lead to the formation of self-assembly networks of DNA and p53 proteins. We propose an aggregation and self-assembly model based on the present observation and discuss its biological meaning.

## 1. Introduction

The tumor suppressor protein p53 is a widely distributed phosphoprotein that is the central player in the pathways controlling cell growth, DNA repair, cell differentiation, senescence, and apoptosis [1,2,3,4,5,6]. It is found that over 50% of human cancers are associated mutations in the p53 protein [1]. The p53 protein is a flexible multidomain protein containing 393 residues in full length, and consists of four distinct domains: (a) the unstructured N-terminal trans-activation domain (N-ter) and a proline-rich domain, which can bind to a series of proteins and regulates p53 transcription [7] and dissociation from DNA [8]; (b) the central core region known as the DNA-binding domain (DBD), which regulates the specific binding to DNA [9]; (c) the tetramerization domain (Tet) and (d) the C-terminal domain (C-Ter) which has been shown to bind nonspecifically to DNA [1,10]. p53 DBD is a sequence-specific transcription factor which is activated in response to a variety of DNA damaging agents, and p53 signaling may suppress apoptosis and induce senescence [5,6]. Many studies have shown that all of these presently known biological functions of p53 critically depend upon its DNA-binding properties in checking the DNA replication stage.

The exact mechanism of p53 binding and its effect on the DNA conformation is still not fully understood. However, it has been found that the p53 multimer, oligomerization, and their stacking can promote a DNA looping reaction [11,12,13]. For example, a tetramer of p53 makes DNA looping easier than using its dimer, while the monomer is unable to form any DNA loops. There are four scenarios that have been proposed: (a) the 3D diffusion mode. In this mode, a protein binds to DNA nonspecifically. The protein dissociates from DNA and subsequently re-associates with another site in the DNA using 3D diffusion [14]. And, the C-Ter and Tet are of critical importance for this mode. They contribute to the fast, nonspecific association of p53 and DNA; (b) The 1D sliding mode. In this mode, the protein remains in contact with the DNA for long periods of time along the DNA chain so that protein diffusive motion occurs [15,16]. Furthermore, the rotation-coupled diffusion motion of p53 along DNA was discovered by simulation [17] and experiment [18,19]; (c) The ‘hopping’ and ‘jumping’ mode. Hopping is repeating transient dissociation and re-association at only one DNA site [20]. Jumping is repeating dissociation and re-association from one DNA site to another or from one port to another port on a single DNA stretch [2,21,22]; (d) The intersegmental transfer mode. In this mode, the protein relocates from one site of DNA to another site by heterotrimeric complex formation [14]. It can elucidate the binding with condensed DNA. In addition, numerous theoretical and experimental investigations have shown that in scenarios where there is a vast excess of accessible DNA to which DNA binding proteins have nonspecific affinity, the protein search process can be efficient if they alternate rounds of 1D sliding while binding nonspecifically and rounds of three-dimensional (3D) diffusion between different sections of DNA [23,24,25,26]. The p53–DNA binding can be a model system to shed light on protein binding mechanisms since it is a key protein subject to facilitated diffusion, where both the 3D diffusion and 1D sliding of p53 have been demonstrated by single-molecule fluorescence microscopy [14,19].

The p53–DNA binding is influenced significantly by the presence of metal ions. Some toxic metal ions (i.e., mercury, cadmium, copper) bind to DNA, resulting in disruption of the p53 conformation and the DNA-binding activity. Some other metal ions, such as magnesium and zinc ions, play an important role in p53 and DNA interactions for stabilizing the structure of the protein and enhancing its affinity to DNA. For example, zinc coordination is thought to be necessary for the proper folding of the p53 core domain, and absence of zinc greatly reduces or abrogates p53–DNA binding and transactivation of target genes [27,28]. Magnesium ions could bind to the p53 DBD protein and enhance the DNA non-specific binding affinity of the p53 DBD [29,30]. Magnesium ions also stabilize the structure of the protein [29]. Studies with cultured cells and animal models have shown that magnesium ions can influence p53 expression and have impact on the tumor suppressing function of p53, hence tumorigenesis [31]. The results are consistent with the extensive computational studies [32].

Atomic force microscopy (AFM) is a convenient tool in biological application since it works in air or liquid conditions which enables it to measure native biological samples in physiological-like conditions [33,34]. Meanwhile, it allows for investigation of structural, mechanical, chemical, and functional properties of many biological samples ranging from DNA, RNA, and protein, to subcellular structures dynamically at single-molecule level [35]. In numerous studies, AFM imaging has been applied to demonstrate protein-induced changes in DNA morphology [36,37,38,39]. It is also a powerful technique for imaging protein–DNA complexes at high resolution [40,41]. For example, it revealed the dynamic interaction between p53 and DNA in solution [2], and provided detailed information on the binding efficiency and spatial distribution of p53 along DNA [1].

In the present study, we imaged the p53–DNA complex at various p53 concentrations and ion conditions to investigate the influence of metal ions on the interaction between p53 and DNA. We found that divalent magnesium ions significantly stimulated the binding of the protein to DNA in a sequence-independent manner, and high concentrations of magnesium ions can promote p53 aggregation and lead to the self-assembly networks of DNA and p53 proteins. The results support the facilitated diffusion mechanism of p53–DNA binding in a mode of combined 3D diffusion and 1D sliding.

## 2. Results and Discussion

### 2.1. AFM Imaging of DNA and p53 Protein Samples

**Figure 1:**
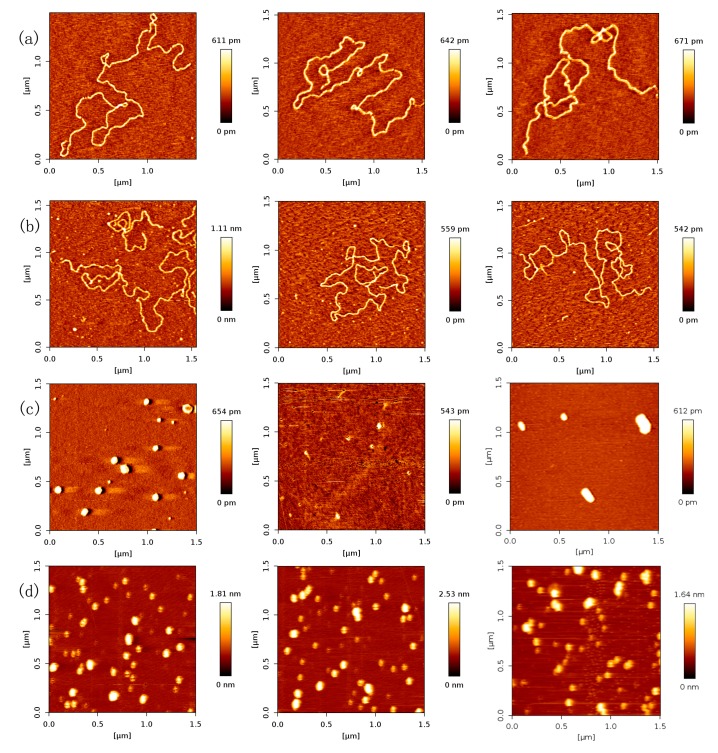
Atomic force microscopy (AFM) images of DNA and p53 (height images, 1.5 µm × 1.5 µm). (**a**) AFM images with control DNA molecules in relaxed conformations, respectively. The environment of the solution is 5 mM Hepes, 3 mM MgCl_2_, pH 7.5, and the concentration of DNA is 1 ng/µL; (**b**) AFM images with control DNA molecules in relaxed conformations, respectively. The environment of the solution is 5 mM Tris, 3 mM MgCl_2_, pH 7.5, and the concentration of DNA is 1 ng/µL; (**c**) AFM images with control p53 molecules in white dot conformations, respectively. The environment of the solution is 5 mM Hepes, 3 mM MgCl_2_, pH 7.5, and the concentration of p53 is 2 ng/µL; (**d**) AFM images with control p53 molecules in white dot conformations, respectively. The environment of the solution is 5 mM Tris, 3 mM MgCl_2_, pH 7.5, and the concentration of p53 is 2 ng/µL.

In previous study of p53 and DNA interaction, Tris and Hepes are usually used as the buffers [2,42,43]. Thus, we performed the imaging of 20,000 bp DNA incubated in the buffer of Tris and Hepes, respectively, with AFM. The AFM images of free DNA are shown in Figure 1a,b, in which DNA exhibited similar uniformly-spread relaxed conformations. Despite some variability in the amount of DNA remaining on the mica surface after rinsing, the morphology of the observed DNA was consistent among all images. The uniform spread of DNA molecules on the mica can be attributed to overall repulsive interactions among the negatively charged DNA molecules, as reported previously [44].

Protein p53 was prepared in the same buffer as the DNA samples and thus contained Mg^2+^ ions as an essential component needed for DNA to adsorb to mica surface. AFM images of p53 revealed small dots that most likely represented monomers of p53 consistent with the size characterization performed on p53 and DNA preparations prior to AFM imaging (shown as Figure 1c,d). We assigned monomer dots by measuring their sizes. The measured diameter and height of monomers are about 24 nm which is consistent with the literature, such as [45]. We can see that the diameter by AFM is larger than the real size of a p53 monomer (~5 nm), because of the cantilever curvature, while the height is quite close to the real quantity. The larger, irregularly-shaped signals most likely represent protein multimers (aggregated forms of p53).

### 2.2. Dependence of p53–DNA Bindings on the Protein Concentration

**Figure 2:**
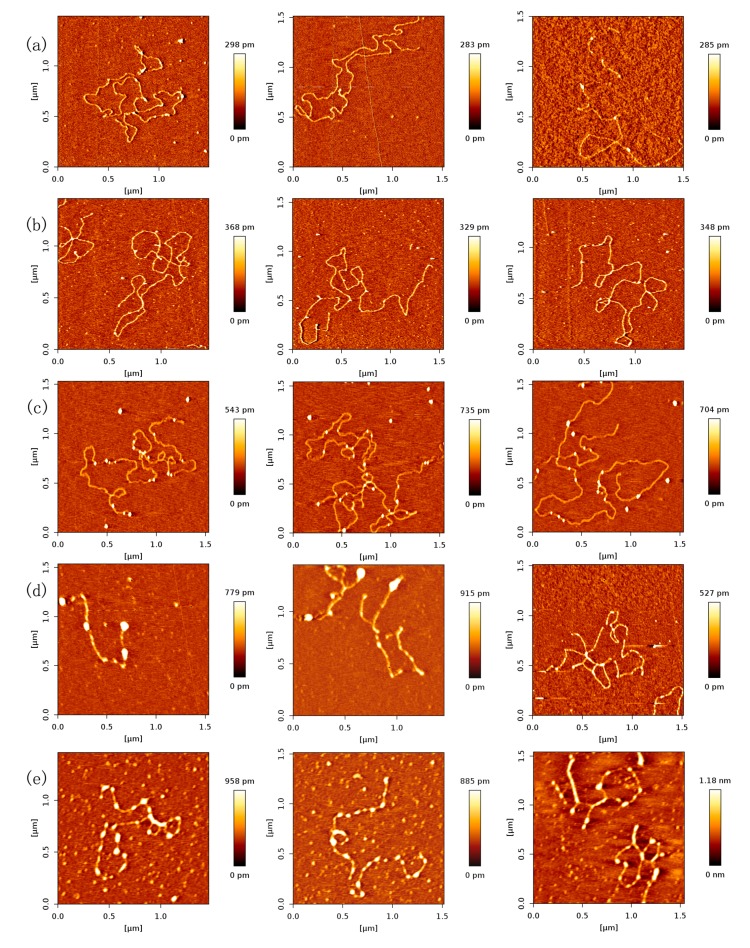
AFM images of p53 and 20,000 bp DNA (height images, 1.5 µm × 1.5 µm), when the concentration ratio of p53:DNA is 1:1, 1.5:1, 2:1, 2.5:1, 3:1, respectively. Meanwhile, the concentration of p53 and DNA are (**a**) 1 and 1 ng/µL; (**b**) 1.5 and 1 ng/µL; (**c**) 2 and 1 ng/µL; (**d**) 2.5 and 1 ng/µL; (**e**) 3 and 1 ng/µL, respectively. The environment of the solution is 5 mM Hepes, 3 mM MgCl_2_, pH 7.5.

In order to investigate the effect of protein concentration, we obtained AFM images of various concentrations of p53 at a fixed concentration of DNA in solution. As expected, we found that the number of p53 proteins binding to DNA grew with increasing concentration of p53 protein, which is shown in Figure 2. From Figure 2a–e, we fixed the concentration of DNA to 1 ng/µL and increased the concentration of p53 from 1 to 3 ng/µL in increments of 0.5 ng/µL.

**Figure 3:**
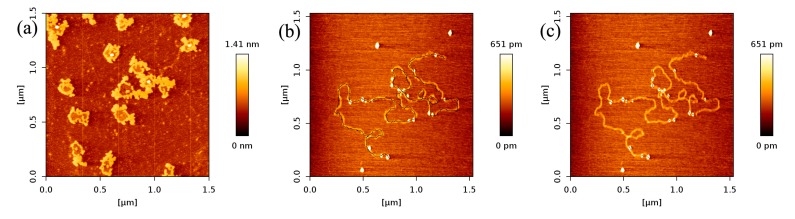
(**a**) AFM images of p53 and 20,000 bp DNA (height images, 1.5 µm × 1.5 µm), when the concentration of p53 and DNA are 4 and 1 ng/µL, respectively; (**b**),(**c**) are DNA length and p53 size measure with image J Software, respectively.

In Figure 2, we can see that the number of p53 proteins binding to DNA increase monotonically with increasing concentration of p53 in solution. Meanwhile, it is notable that the heights of p53–DNA complexes on the mica surface elevate slightly from Figure 2a–e: from 0.289 ± 0.008 nm (a); to 0.348 ± 0.019 nm (b); to 0.660 ± 0.103 nm (c); to 0.740 ± 0.196 nm (d) and to 1.007 ± 0.153 nm (e). When the concentration of p53 in solution continues to increase to some threshold, we can find the phenomenon of saturation, which means all binding sites of DNA are occupied by p53. The threshold value is about 4 ng/µL when the concentration of DNA is fixed to1 ng/µL, which is shown in Figure 3a, where p53–DNA complexes form many loops. On the other hand, we can quantitatively measure DNA length and p53 size with equipped image analysis software. For example, as shown in Figure 3b,c, we obtained that the contour length of 20,000 bp DNA was 6.07 ± 0.209 µm, and p53 size was 0.022 ± 0.0047 µm. Therefore, we estimated that the maximum number of 20,000 bp DNA binding site occupied by p53 is 276, approximately, which corresponds to 45 proteins per µm DNA.

Based on a large number of AFM images of p53–DNA complexes, we obtained the mean values of number of p53 proteins for 1 µm DNA at various concentration p53, listed in Table 1. From the values, we fitted the logistic function about the dissociation constant *K*_d_ by the least-squares method, and found that its value is 328.02 nmol/L. In the same ion condition, the *K*_d_ of the monomer is much more than the tetramer’s, in other words, the binding affinity of monomer is much less than the tetramer’s. This might be partly the reason for transcriptional activation by the p53 tetramer rather than the monomer in cells.

**Figure 4:**
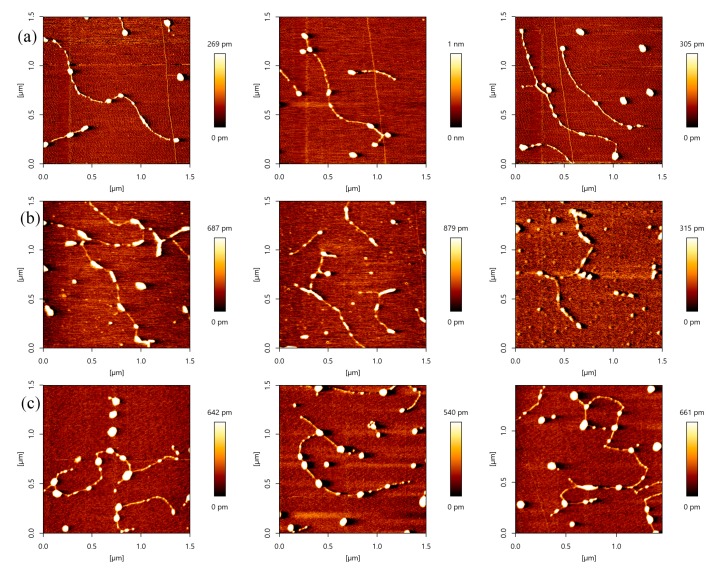
AFM images of p53 and 5000 bp DNA (height images, 1.5 μm × 1.5 μm), when the concentration ratio of p53:DNA is 1:1, 2:1, 3:1, respectively. Meanwhile, the concentration of p53 and DNA are (**a**) 1 and 1 ng/µL; (**b**) 2 and 1 ng/µL; (**c**) 3 and 1 ng/µL, respectively. The environment of the solution is 5 mM Hepes, 3 mM MgCl_2_, pH 7.5.

To solidify the findings from the experiment above, we replaced 20,000 bp DNA with 5000 bp DNA and repeated the experimental procedure. The results are shown in Figure 4, which shows the same tendency of the quantities of p53 binding to 20,000 bp DNA. The mean values of the number of p53 proteins adhering to 1 µm DNA at various concentration p53 are listed in Table 2, corresponding to an approximate same *K*_d_ as the results of p53 and 20,000 bp DNA.

### 2.3. p53–DNA Binding in the Influence of Magnesium Ions

**Figure 5:**
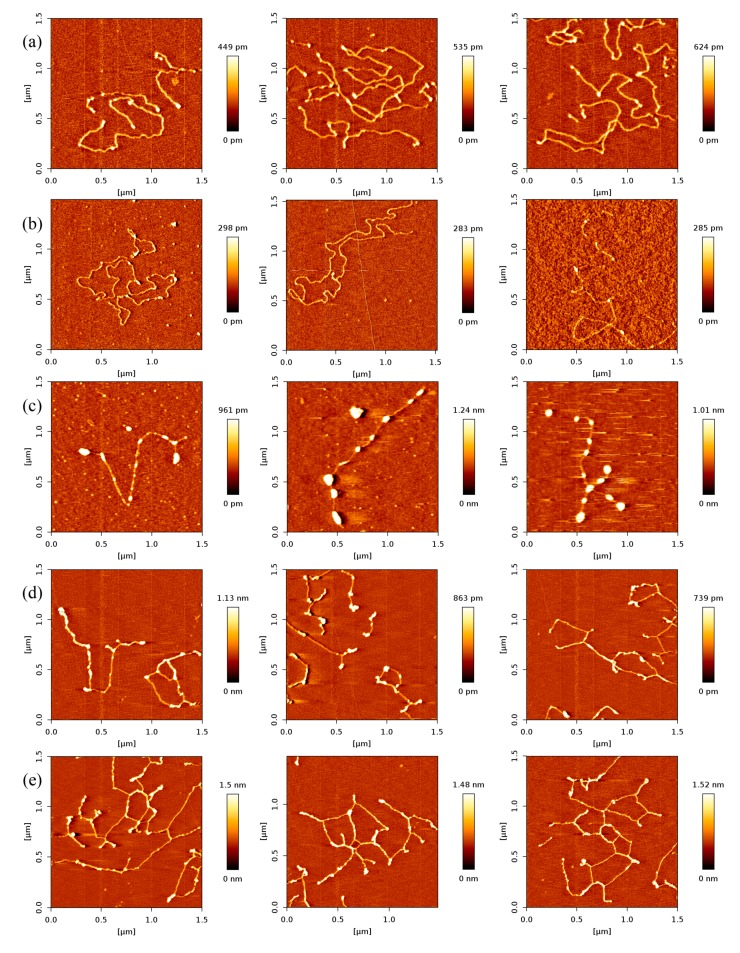
AFM images of p53 and 20,000 bp DNA, when the concentration ratio of p53 and DNA is 1:1 (height images, 1.5 μm × 1.5 μm). Meanwhile, the concentrations of p53 and DNA are 1 ng/µL and 1 ng/µL, respectively. The environment of the solution is 5 mM Hepes, pH 7.5 containing (**a**) 1 mM MgCl_2_; (b) 3 mM MgCl_2_; (**c**) 5mM MgCl_2_; (**d**) 8 mM MgCl_2_; (**e**) 10 mM MgCl_2_, respectively.

Magnesium ions have significantly influenced the p53 sliding on DNA. When the concentration of Mg^2+^ is low, i.e. 0–3 mM, the concentration of Mg^2+^ would regulate the velocity of p53 sliding on DNA. When the concentration of Mg^2+^ increases, the velocity of p53 sliding on DNA would increase clearly [43]. In the present AFM investigation of magnesium ion effect on p53–DNA binding, we fixed both concentrations p53 and DNA to 1 ng/µL. The AFM images of p53–DNA complexes in solutions of different magnesium ion concentrations are shown in Figure 5. Before the complexes were adsorbed on mica surfaces, they were incubated in the solution containing 1, 3, 5, 8 and 10 mM MgCl_2_, respectively, the corresponding images are shown in Figure 5a–e. From Figure 5a–c, we can see that the number of p53 proteins binding to DNA increases with the concentration of magnesium ions. On the other hand, we noticed that the size of some p53–complexes grows simultaneously with Mg^2+^ concentration even though the concentration of p53 is fixed. From this observation, we deduce that magnesium ions enhance the nonspecific binding of p53 to DNA, resulting in the increasing number of p53–DNA complexes, and/or p53 itself aggregating, indicated by the growing size of some complexes.

When we increase the concentration of Mg^2+^ further, some transitions happen. For example, when the solution contained 8 mM Mg^2+^ (or 10 mM Mg^2+^), we found that some p53–DNA complexes condensed and aggregated (shown as Figure 5d or Figure 5e). The agglomeration degree of p53–DNA complexes was enhanced as the concentration of magnesium increased. This can be explained as follows: magnesium ions weaken the binding of p53 to DNA and accelerate its sliding along DNA. Meanwhile, the sliding proteins aggregate along DNA and self-assemble to form DNA–p53 network structures. By volume estimation, we infer that one aggregation usually consists of at least 3 monomers and up to more than 20. In the 5-mM magnesium ion condition, many p53 proteins form a large aggregate. When the ion concentration increases further, p53 proteins assemble along DNA and the network structures forms, appearing smaller in lateral dimension. Therefore, p53, adjusted by magnesium ions, induced DNA condensation occurrence and p53–DNA self-assembly. p53 also promotes DNA to form networks when the concentration of Mg^2+^ was more than 8 mM. In previous works, Mg^2+^ was thought to be unable to facilitate DNA condensation and aggregation. The effect of Mg^2+^ on p53–DNA interaction has not been systematically determined, even though it has been proposed that the p53 core domain was rarely observed to adhere to supercoiled DNA at a Mg^2+^ concentration higher than 0.5 mM [43]. Thus, we deduced that Mg^2+^ may enhance the p53–DNA aggregation and self-association property. These images provide new insights about p53–DNA interactions and the effect of divalent cation on p53–DNA interaction. We focus on the non-specific binding to DNA and aggregation of p53, similar with those of Reference [13]. The reference demonstrated that p53 causes looping of DNA by connecting multiple regions of the relaxed DNA, while we emphasize the influence of magnesium on p53 aggregation and self-assembly along DNA.

Molecular self-assembly is a powerful approach to fabricate supramolecular architectures. Self-assembled structures on surfaces have been made from hydrogen-bonded systems [46]. Our results suggest p53 may regulate DNA self-assembly to inhibit oncogene occurrence.

**Figure 6:**
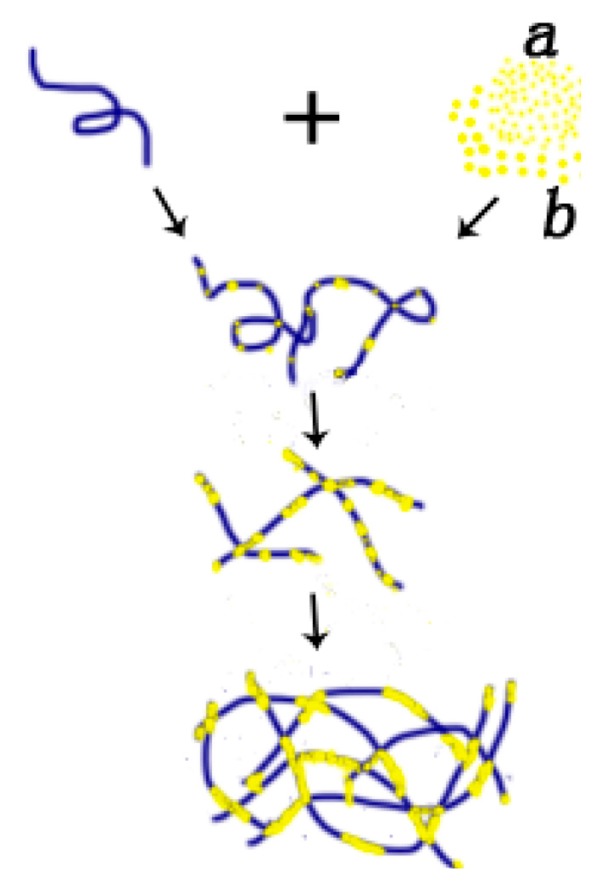
Schematic for p53–DNA binding. (**a**) Firstly, some p53 molecules spontaneously adhere to one DNA molecule by Brownian movement or electrostatic interaction. Secondly, the other free p53 molecules integrate with some p53 molecules adhering on one DNA, under a certain magnesium effect. And, the other free p53 molecules integrate with p53 adhering on one DNA, repeatedly. Thirdly, some p53 molecules adhering to DNA connect with the other p53 adhering to DNA, forming large-scale networks; (**b**) Initially, some p53 molecules integrate with other p53, under the magnesium effect. After that, some p53 aggregations adhere to one DNA molecules. Finally, p53 makes it possible to spontaneously link p53 under the magnesium effect, forming large-scale networks, repeatedly. Note that: (**a**) is related to the top part of protein (small yellow beads in the right of first line); (**b**) is related to the bottom part of protein (big yellow beads in the right of first line).

Based on the above observations, we can construct a feasible model of p53–DNA binding with the influence of magnesium ions, which is shown in Figure 6. The binding process consists of two steps: (a) some p53 spontaneously associates non-specifically to a segment of DNA by Brownian movement or electrostatic interaction. In the presence of magnesium ions, free p53 proteins in solution integrate with those already adhered to DNA, repeatedly; (b) Meanwhile, p53 can attract each other and aggregate to form a larger particle under the magnesium ion effect. The larger p53 aggregations can also bind a DNA segment in a non-specific manner, as we have seen in Figure 5b,c. In the solution with a high concentration of magnesium ions, the p53–DNA complexes link each other to spontaneously form a self-assembly structure, resulting in large-scale networks, as shown in Figure 5d,e. The binding and aggregation might also occur simultaneously.

Although our measurements were completed in vitro, the results may have some bearing in vivo, especially for the relation between the aggregation of p53 and tumor occurrences. The total concentration of Mg^2+^ in mammalian cell ranges between 14 and 20 mM, and the concentration of free Mg^2+^ is 0.5–0.7 mM [47]. The free part of Mg^2+^ has no significant effect on p53 aggregation. However, p53 aggregation might occur if most magnesium ions release in some extreme conditions. Our experiments show that the self-assembly of p53–DNA not only depends on the concentration of p53, but also on the existence of magnesium ions. Magnesium ions in solution promote the binding of p53 to DNA non-specifically to form many p53–DNA complexes. It has been shown that magnesium ions can accelerate p53 sliding on DNA in a manner of Brownian motion under conditions of high magnesium ion concentration [43]. When two complexes collide along DNA, they form an aggregation, implying a process of nucleation-growth. The procedure repeats and finally a network structure of p53–DNA forms. An alternative mechanism is that p53 binds to two parts of DNA and forms a higher order of aggregation. In conditions of high magnesium ion concentration, even if p53 concentration is relatively low, p53 and DNA are still able to aggregate and self-assemble. Actually, there is strong proof for the intracellular aggregation of mutant p53 in cancers [48,49]. p53 aggregation promotes tumor progression and affects a variety of basic functions of RNA transcription, translation, and the cytoskeleton. Furthermore, inhibition of p53 aggregation can activate the pro-apoptotic function of the p53 protein. In addition, p53 aggregation can promote chemoresistance, and has been used as a new marker for chemoresistance [50]. Therefore, we infer that high concentrations of magnesium ions may be related with the occurrence of tumors.

## 3. Materials and Methods

### 3.1. DNA and Human p53 Protein

Plasmid DNA (20,000 bp/5000 bp) (Fermentas/Thermo Fisher Scientific, Burlington, ON, Canada) was purchased from Thermo Scientific and used without further purification. As received from the manufacturer, the 20,000 bp/5000 bp DNA stock solution had a concentration of 500 ng/µL. The solvent was 1× TE buffer, which was composed of 10 mM Tris-HCl, pH 7.6, and 1 mM EDTA. Human p53 protein was purchased from Sigma-Aldrich (St. Louis, MO, USA), and the concentration of which was 200–600 ng/µL. The p53 stock solution contained 50 mM sodium phosphate and 50 mM NaCl in pH 7.5. The p53 human-recombinant protein was expressed in *Escherichia coli* and purified by two chromatographic steps: affinity chromatography and gel filtration chromatography. And, the final purified p53 was checked on an SDS-PAGE with more than 90% purity. The DNA binding activity of p53 was examined using an electrophoretic mobility shift assay.

Water was deionized and purified by a Millipore system (Millipore Corporation, Billerica, MA, USA) and had a conductivity less than 1 × 10^−6^ Ω^−1^·cm^−1^. Mica adsorbing DNA and p53 proteins for AFM imaging was cut into approximately 1.0–1.5 cm^2^ square pieces and their surfaces were always freshly cleaved before use. Other chemical agents were all purchased from Sigma-Aldrich and used as received.

The pH value of ions and buffer solution was adjusted by adding hydrochloric acid to the stock buffer, and were measured by a Sartorius Basic pH meter PB-10 (Sartorius AG, Gottingen, Germany).

### 3.2. AFM Sample Preparation

#### 3.2.1. DNA and p53 Samples

The DNA samples were prepared by incubating DNA (at the initial stock concentration of 500 ng/µL) in 5 mM Hepes buffer with 3 mM MgCl_2_ (pH 7.5). The addition of divalent ions, such as Mg^2+^, to the buffer helps is to help the negatively charged DNA adsorb to mica surfaces [37,44]. DNA samples of 200 µL and of concentration 1 ng/µL containing 5 mM Hepes and 3 mM MgCl_2_ (pH 7.5) were incubated in ice for more than 30 min in fridge at 0 °C. After incubation, 30 µL of the DNA solution was deposited on a freshly cleaved mica surface (1 cm × 1 cm), and incubated for 3 min to allow DNA to adsorb to the mica substrate. The mica surface with DNA was lightly rinsed about 15 times with 50 µL of deionized water to remove excess molecules and subsequently dried with a gentle nitrogen flow prior to AFM imaging.

The protein samples with p53 protein were prepared from stock concentrations of 200–600 ng/µL, using the same buffer that was used in preparation of the DNA samples to dilute to protein concentrations in the range of 1.6–2.4 ng/µL before AFM imaging. The sample preparation of p53 for AFM imaging follows the similar protocol as for DNA.

#### 3.2.2. p53–DNA Complex Samples

The p53–DNA complex samples were prepared by incubating stock solutions of p53 protein with the DNA stock solution in the same buffer for DNA and protein. Briefly, 0.8 µL of p53 protein and 0.4 µL of DNA stock solutions were mixed in 198.8 µL buffer containing 5 mM Hepes and 3 mM MgCl_2_ (pH 7.5) to form 200 µL of p53–DNA solution. The final concentrations of p53 protein and DNA in the solution were 1.6–2.4 and 1 ng/µL, respectively. Experiments were performed by mixing different amounts of p53 with the DNA solution. The samples were incubated in ice at 0 °C for more than 30 min prior to depositing onto freshly cleaved mica surfaces. A drop of 30 µL of the p53–DNA solution was used to deposit on mica surface for 3 min, and then the surface was gently rinsed about 15 times with 50 µL of deionized water to remove excess molecules and subsequently blown dry with a gentle nitrogen gas.

### 3.3. AFM Imaging of p53–DNA Complexes

After placing in air for 2–4 h, the prepared samples were scanned by AFM (JPK Nano Wizard III, Berlin, Germany) in AC mode. A 125 µm-long and 30 µm-wide and 4 µm-thickness silicon AFM probe with aluminum coating, spring constant 42 N/m, and resonance frequency of 320 kHz (NCHR-50, NanoWorld Corporation, Neuchâtel, Switzerland) was used. All images were captured from a 1.5 µm × 1.5 µm viewing area on the sample by a scan rate of 1.0 Hz. Each image was 512 × 512 pixels (4–6 nm/pixel). For each sample, 3–10 images were acquired from different regions within it.

Raw images were pre-processed by the equipped JPK Data Processing Software version 4.2 (JPK AG, Berlin, Germany) to eliminate the drift and to normalize the data [51]. Measurements of the morphologies observed on the final digitized images were performed with the same Software [2]. About 20 DNA molecules were analyzed for each mica sample from different 1.5 µm × 1.5 µm areas. We then counted the number of p53 molecules binding to DNA in different concentration ratios of p53:DNA in AFM images and obtained the mean values in corresponding to concentration ratios. The variance between the twenty samples did not exceed 20%.

## 4. Conclusions

In summary, we have investigated the influence of magnesium ions on the interaction between p53 and DNA. We found that Mg^2+^ significantly stimulates the binding of the protein to DNA in a sequence-independent manner, different from that of zinc ions, which is in a sequence-specific manner. We also found that a high concentration of magnesium ions can promote p53 aggregation and the formation of self-assembly networks of DNA and p53 proteins. From our observations, we surmise that a high concentration of magnesium ions might be an affecting factor in the occurrence of cancers.

## Figures and Tables

**Table 1:** The mean quantities and probability of p53 adhering to 1 µm DNA, corresponding to the concentration ratios of p53 and DNA.

The Concentration Ratio of p53 and DNA	p53 Molar Concentration (nmol/L)	The Quantity of p53 Adhering to DNA (/μm DNA)	Probability of DNA Binding Site Occupied by p53
0	0	0	0
1:1	18.867	0.8 ± 0.2	0.018 ± 0.005
1.5:1	28.3005	1.6 ± 0.5	0.036 ± 0.011
2:1	37.734	2.9 ± 0.6	0.064 ± 0.013
2.5:1	47.1675	4.9 ± 0.9	0.11 ± 0.021
3:1	56.601	10.2 ± 1.6	0.23 ± 0.037

**Table 2:** Mean quantities and probability of p53 adhering to 1 µm 5000 bp DNA, corresponding to the concentration ratios of p53 and 5000 bp DNA.

The Concentration Ratio of p53 and DNA	p53 Molar Concentration (nmol/L)	The Quantity of p53 Adhering to DNA(/μm DNA)	Probability of DNA Binding Site Occupied by p53
0	0	0	0
1:1	18.867	4.2 ± 1.8	0.093 ± 0.040
2:1	37.734	6.2 ± 4.9	0.137 ± 0.110
3:1	56.601	6.3 ± 3.8	0.139 ± 0.084
